# A Framework for the Evaluation of Biosecurity, Commercial, Regulatory, and Scientific Impacts of Plant Viruses and Viroids Identified by NGS Technologies

**DOI:** 10.3389/fmicb.2017.00045

**Published:** 2017-01-24

**Authors:** Sebastien Massart, Thierry Candresse, José Gil, Christophe Lacomme, Lukas Predajna, Maja Ravnikar, Jean-Sébastien Reynard, Artemis Rumbou, Pasquale Saldarelli, Dijana Škorić, Eeva J. Vainio, Jari P. T. Valkonen, Hervé Vanderschuren, Christina Varveri, Thierry Wetzel

**Affiliations:** ^1^Plant Pathology Laboratory, Gembloux Agro-Bio Tech, University of LiègeGembloux, Belgium; ^2^Institut National de la Recherche Agronomique (INRA), University of Bordeaux, CS20032 UMR 1332 BFPVillenave d'Ornon, France; ^3^Plant Biology, Linnean Centre for Plant Biology, Uppsala BioCentre, Swedish University of Agricultural SciencesUppsala, Sweden; ^4^Virology and Zoology, Science and Advice for Scottish AgricultureEdinbourgh, UK; ^5^Department of Plant Virology, Institute of Virology, Biomedical Research Center, Slovak Academy of Science (SAS)Bratislava, Slovakia; ^6^Department of Biotechnology and Systems Biology, National Institute of BiologyLjubljana, Slovenia; ^7^Virology, AgroscopeNyon, Switzerland; ^8^Division Phytomedicine Lentzeallee, Faculty of Life Sciences, Albrecht Daniel Thaer-Institute of Agricultural and Horticultural Sciences, Humboldt-University of BerlinBerlin, Germany; ^9^National Research Council Institute for Sustainable Plant ProtectionBari, Italy; ^10^Department of Biology, Faculty of Science, University of ZagrebZagreb, Croatia; ^11^Management and Production of Renewable Resources, Natural Resources Institute Finland (Luke)Helsinki, Finland; ^12^Department of Agricultural Sciences, University of HelsinkiHelsinki, Finland; ^13^Plant Genetics, Gembloux Agro-Bio Tech, University of LiègeGembloux, Belgium; ^14^Department of Phytopathology, Benaki Phytopathological InstituteAthens, Greece; ^15^DLR Rheinpfalz, Institute of Plant Protection, Neustadt an der WeinstrasseGermany

**Keywords:** NGS, pest risk analysis, virus diseases, biological characterization, plant health, regulatory agencies

## Abstract

Recent advances in high-throughput sequencing technologies and bioinformatics have generated huge new opportunities for discovering and diagnosing plant viruses and viroids. Plant virology has undoubtedly benefited from these new methodologies, but at the same time, faces now substantial bottlenecks, namely the biological characterization of the newly discovered viruses and the analysis of their impact at the biosecurity, commercial, regulatory, and scientific levels. This paper proposes a scaled and progressive scientific framework for efficient biological characterization and risk assessment when a previously known or a new plant virus is detected by next generation sequencing (NGS) technologies. Four case studies are also presented to illustrate the need for such a framework, and to discuss the scenarios.

## Introduction

Until recently, plant virus discovery appeared as a long and fastidious task, mainly driven by the need to identify the etiology of diseases of unknown origin. In the last few years, however, the advent of next generation sequencing (NGS) has revolutionized the study of plant viruses by providing a powerful alternative for their detection and identification, without any *a priori* knowledge. NGS technologies are progressively reaching the diagnostic field (Massart et al., 2014), impacting also quarantine regulations (Martin et al., 2016). Their use allows the continuous discovery of new plant viruses, the observation of an increasing diversity of variants for known viruses and the frequent existence of a complex of different viruses. Downstream of these findings, the main challenge to be addressed is the biological significance (pathogenicity, hosts, transmission, epidemiology…) of the discovery of a novel virus species or strain in single plants, particularly in asymptomatic plants and for viruses that are the founding members of new virus genera or families and share little or no sequence similarity with known viruses (Wu et al., 2015). Plant virologists are now challenged to do the work backwards, namely to characterize new viruses, with genome sequence information as the unique starting point. Furthermore, the ability of NGS to detect cryptic (symptomless) viruses in cultivated and wild plants, and the reported mutualistic interactions challenging the traditional dogma that all viruses are pathogens (Roossinck, 2011), are adding new difficulties to the prediction of the impact of new viruses.

Undoubtedly, these new scientific outcomes will have to be handled with extreme care in terms of dissemination from basic research to applied agronomy, and to plant health, agriculture and forestry policy makers and regulators. Indeed, the amount of information needed to assess the risk posed by a new virus species to a certain commodity or region is huge. Scientists may indeed have to work for years to provide the answers needed to conduct a thorough Pest Risk Analysis (PRA) according to international phytosanitary standards (ISPM 2 and ISPM 11) (FAO, 2004, 2007). Therefore, the main challenge for scientists arising from the discovery of a new viral sequence is to efficiently characterize the biological properties through efficient short, mid-, and long-term strategies while creating appropriate communication channels with the regulatory authorities. In this paper, we aim to discuss the new challenges raised by NGS as illustrated by case studies, and to suggest guidelines for researchers, policy makers, plant health authorities and plant inspection services by describing the necessary steps, the appropriate interactions and the inherent prioritization to be followed after discovering a new virus sequence [or a new isolate or variant, or (a) new host(s)].

Overall, the advent of new NGS technologies wisely complemented by traditional or classical virus study methods is expected to globally promote plant health and plant protection, but may also cause mayhem in trade and agriculture if challenges and questions are not addressed properly.

## Current situation with known viruses

Quarantine and certification lists are regularly updated following new PRAs. They are based on 6 categories of information: (i) knowledge of the identity of the pest (and therefore being able to differentiate it from other viral agents); (ii) data on its distribution and (iii) host range; (iv) information on the modes and efficiency of spread and on the identity of any vector(s); (v) suitability of the local agro-environmental conditions for the pest [and vector(s)]; and (vi) the ability to cause a disease and impact the development, reproduction, or productivity of cultivated or wild host plants. Further, refinement of the risk assessment can be based on additional information such as the availability of efficient and easy-to-implement control methods.

The PRA provides the risk assessor with a risk assessment tool whose details will depend both on data availability concerning the six points listed above and on the needs of the risk manager, whose role is to consider the available scientific information (risk assessment) as well as other factors (economical, acceptable political risk, feasibility, and impact of measures) in reaching a decision on whether to regulate or address the pest in any specific way.

## Emerging challenges for scientists and authorities

**Figure 1:**
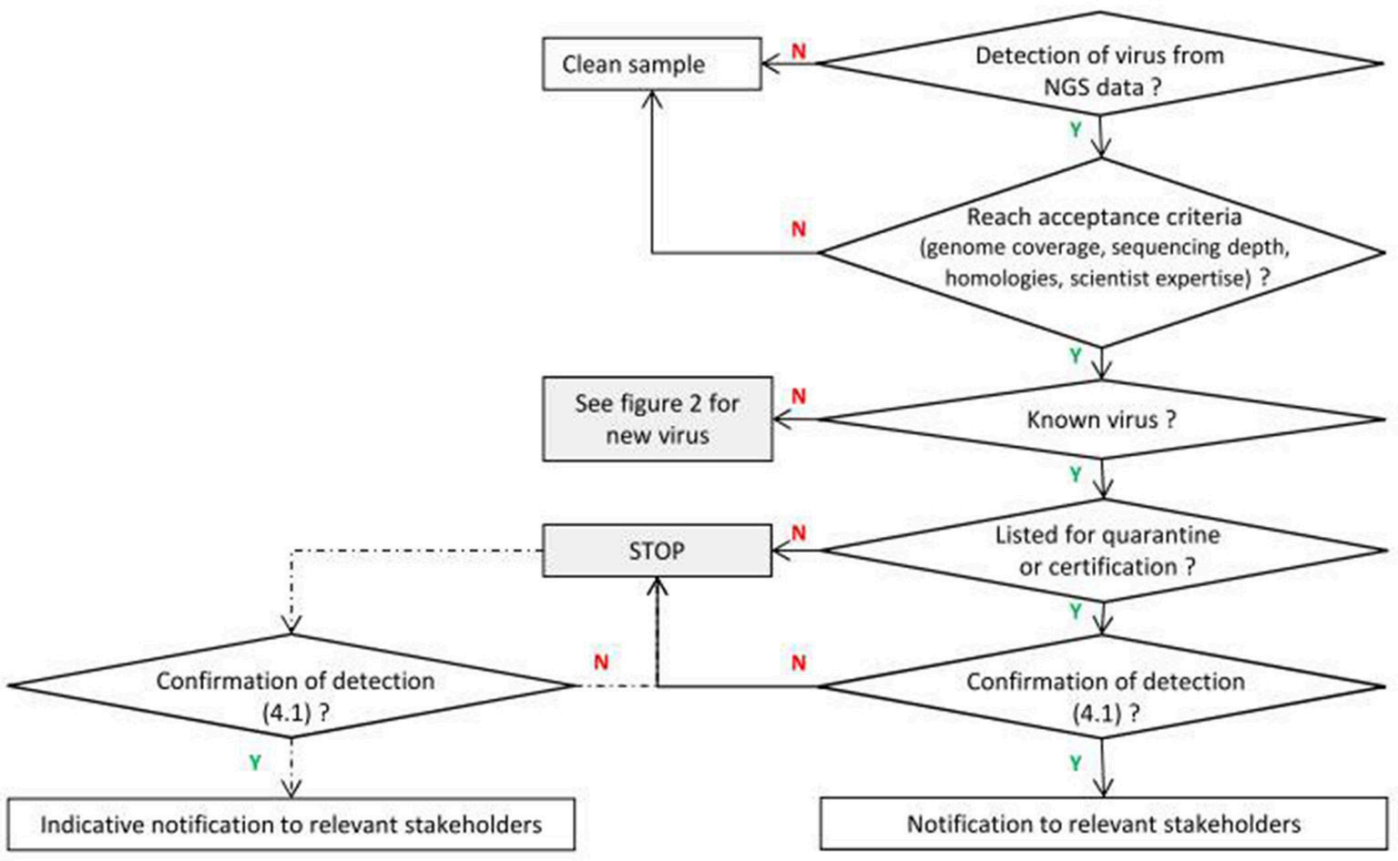
**Framework for known viruses**. Y means positive response and N negative response (dotted lines correspond to optional analyses for non quarantine pathogens depending on the request of the customer).

When a known virus is detected by NGS technologies, the main questions are related to its legal status (see Figure 1). The decision scheme to be applied is based on existing quarantine or certification regulations and is similar to the process routinely carried out with PCR or ELISA-based methodologies. Nevertheless, NGS may also reveal additional complexity and even reorient disease investigation for known species (see the Study Case N°1 in Supplementary Material).

It should be stressed that risk assessment is sometimes needed or requested by National Authorities in situations where hardly any information is available, a seen in recent examples in 2016 for Pepper vein yellow virus (PeVYV) or Grapevine pinot gris virus (Germany). In such a case, risk assessors cannot wait until enough data is available and first conclusions have to be reached based on whatever information is at hand. This must be done cautiously, while simultaneously taking into account the uncertainties associated to or caused by the lack of data and making them clear to the risk manager.

In this context, the increasing identification of new viral sequences by NGS technologies may complicate the decision-making process for certification programs, quarantine processes, and more generally the trading of plant materials (see the Study Case N°2 in Supplementary Material). The only information available may be the full or partial genome sequence with the additional complexity that it may have been detected in asymptomatic samples or in combination with other viruses. It is therefore increasingly important to understand the biology of any new viral sequence to provide a basis for assessing the risk they pose and take scientifically-based decisions. Scientists are therefore now challenged to provide biological data on these newly described viral sequences, in a short timeframe and with limited funding. The following chapters propose a framework of scaled actions to efficiently address the need for PRA of regulatory authorities.

## Early steps of biological characterization

**Figure 2:**
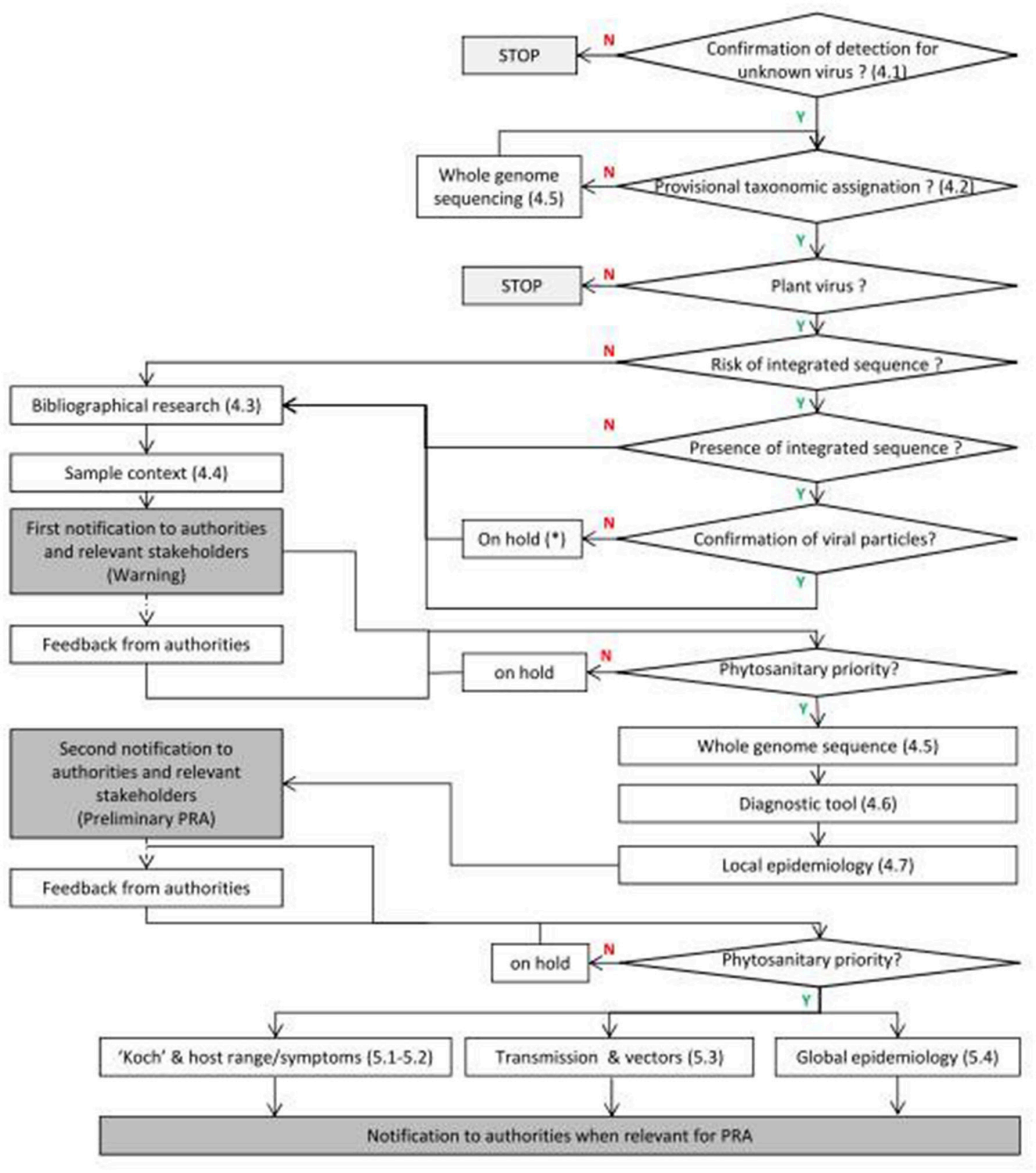
**Framework proposal following discovery of new viruses**. Y means positive response and N negative response. The numbers between brackets correspond to the chapters in the text. ^*^The absence of viral particle should be recorded and further experiments carried out if new cases are discovered.

The detection of a new viral sequence should directly be followed by several immediate actions for its early biological characterization to reach a first decision level of notification to authorities (see Figure 2).

### Confirmation of detection

To dispel a laboratory or bioinformatics error or contamination, NGS-based identification of putative new viral sequence or new strains of existing viral species requires further confirmation by complementary methods, usually by (RT)-PCR using specific primers. If the concentration is very low, the sample can be enriched in viral particles through homogenization, filtration, and/or ultracentrifugation prior to nucleic acid extraction. Enriched viral particles can also be observed by electron microscopy, even if electron microscopy lacks sensitivity and viral particles can be missed. For DNA viruses, especially *Caulimoviridae*, additional research is also needed to evaluate whether the detected viral sequences exist in an episomal form and not only as the trace of a “fossilized” virus integrated in the plant genome (Liu et al., 2010; Aiewsakun and Katzourakis, 2015).

### Provisional taxonomy assignment

The assembled contig will generally allow for provisional taxonomic assignment of the viral sequence. Nevertheless, this can turn out to be a very difficult task, made even more complex by (i) the frequent incompleteness of the genome sequence data obtained, (ii) the variable and irregular coverage of viral genomes, (iii) the incompleteness of viral taxonomy, and (iv) the variability of species, genus, or family molecular discrimination criteria. Specific guidelines can be found in the latest ICTV report (http://www.ictvonline.org). The detected viral sequences may also correspond to a *bona fide* virus infecting other organisms associated with the sample, including bacteria, fungi or arthropods (Al Rwahnih et al., 2011; Marzano and Domier, 2015) or to viral sequences integrated into the plant genome. The future solution to this problem is to establish the baseline of virus presence through an improved knowledge of the viromes of agronomically important plants as well as viromes of natural ecosystems, including the integrated viral sequences.

### Bibliography

Overall, provisional taxonomic assignment should always be considered with caution but can give first clues on the physical and/or chemical properties of a virus as well as on its biology, including (i) the putative host range for the tentative identification of alternative hosts, (ii) the prediction of the pathogenic potential in those hosts, (iii) the modes of horizontal and/or vertical transmission, including the identification of candidate vectors. These hypotheses have practical consequences such as the selection of appropriate hosts and experimental modes of inoculation, a first assessment of the possible epidemic potential of a new viral agent, or a tentative identification of possible alternative hosts in ecosystems. Altogether, this early step, which requires expertise in molecular biology, in plant virology, and basic bioinformatics skills, might lead to the elaboration of a draft epidemiological scenario (Loconsole et al., 2012; Adams et al., 2014). Nevertheless, this approach can be biased as for example in the genus *Closteroviridae*, which includes economically important viruses with similar virion morphology, genome size, and organization but with contrasted biological properties (Martelli et al., 2012).

### Sample documentation

In addition, all the metadata about the sample should be collected precisely to feed the risk assessment process. It is important to document symptoms (if any) and time of appearance, to identify the plant species and cultivar, to describe the sampled plant part (leaves, fruit, seeds, etc.), the geographical origin of the sample, its intended use etc…Any missing information should be completed at this stage so as to be included in the first notification.

### Full genome sequencing and annotation

The assembly of viral sequence(s) based on NGS reads can result in three scenarios of genome coverage: (i) complete, (ii) incomplete but continuous, or (iii) a set of scaffolds and contigs. The reconstruction of the full genome sequence of a candidate new virus is mainly based on targeted (RT)-PCR amplification and sequencing to fill the sequence gaps between *de novo* generated contigs, verify their exclusive viral origin and the absence of host-derived sequences. Taxonomic assignment can also be fine-tuned thanks to the whole genome sequence; but in a worst case situation, even the availability of a complete genomic sequence may be insufficient to settle such a question (Marais et al., 2016).

### Development of a diagnostic protocol

Identifying a new virus species, even from a partial genome sequence, triggers the immediate need to develop a diagnostic protocol. This is a fundamental step toward the management of viral diseases in cultivated crops or the unveiling of a new virus eco-epidemiological role in wild plants. The diagnostic of newly identified and confirmed viruses is usually done using PCR-based methods (the most popular ones for new viruses) or with the LAMP technology, as a recent alternative (Boonham et al., 2014). Nucleic acid-based methods are currently preferred to diagnose new viruses because they are quicker, easier and cheaper to develop than antibody production for ELISA.

### Field/batch observation

Small-scale epidemiological surveys at the discovery location can be undertaken. Such surveys will also take into account the hypotheses that may have arisen after bibliographical research and should be focused on the plant species where the virus was detected; but they could also take into account, whenever possible, the potential host(s) and vector(s), their geographical spread and seasonality. Likewise, sampling methods enabling for the statistical linking of findings and field observations should be selected. Several scenarios are possible depending on the origin of the sample: field or trade/quarantine/certification.

The presence of symptoms in the initial field host has often been perceived as a starting point for further investigations about the presence of a virus, but asymptomatic plants must also be included in the sampling because symptomatology reflects the complex interplay of infectious agent(s), the host plant metabolism, its defense systems and varietal specificities, as well as abiotic factors. The advent of wide metagenomic studies of environmental viromes (Kristensen et al., 2010; Mohiuddin and Schellhorn, 2015) allows for the identification of new viruses from any sample, including asymptomatic field samples. Thus, even if focused symptom observations in the field cannot be neglected, a survey cannot be led by symptomatology alone. Hence, virus spread to other agro-ecological niches where they may encounter new hosts, co-infecting viruses or vectors, may cause a shift in their pathogenic potential and result in a serious disease.

## In-depth biological characterization

The in-depth biological characterization of a new virus is envisioned as a mid- to long-term goal in order to gradually reduce the uncertainties associated with the early risk assessment and decision-making processes. More specifically, in-depth biological characterization aims to build comprehensive knowledge on the symptomatology/etiology, epidemiology and ecology of a new virus by gathering information on its host range, its symptomatology on various cultivars and host species, its vectors and modes of transmission, its geographical distribution, and its interactions with other viruses. Fulfilling Koch's postulates can be considered as a cornerstone and is pivotal in this in-depth biological characterization. An additional difficulty arises in the case of a virus identified from asymptomatic tissue or viruses with potentially neutral of beneficial interactions with the plants (Xu et al., 2008; van Molken et al., 2012). In these cases, Koch's postulates may prove useless, because they presume the development of disease symptoms following introduction of the pathogen into a healthy host. But laboratory and greenhouse experiments as well as field surveys will bring more information and might bring the proof of mutualistic or synergistic interactions between the virus and the plant. These data will be necessary for risk assessment to make further progress by determining whether the virus is present (locally or widespread) or not in a given geographical area, its potential for spread (type of transmission, host range), and ultimately to evaluate its economic impact (symptoms alone or in association). In turn, local authorities may define appropriate regulatory measures in relation to Plant Health protection and control if required. The Study Case N°3 on *Grapevine Pinot Gris virus* (see Supplementary Material) exemplifies this complex approach.

### Transmission experiments

The inoculation of a new virus to test plants in laboratory/greenhouse conditions is the basis for any biological characterization and may be particularly complex. Depending on the objective, the virus can be transmitted to indicator plants, host plant candidates or other cultivars of a same species. The modes of vertical and horizontal transmission of viruses are diverse, so that the integration of taxonomic (Figure 2 and Section Provisional Taxonomy Assignment), bibliographical (Figure 2 and Section Bibliography), and field survey data (Figure 2 and Section Field Surveys) is of utmost importance for the proper design of experimental transmission procedures in the laboratory or the greenhouse.

Plants can be inoculated by several techniques, the most common ones being mechanical inoculation, grafting, vector inoculation or the use of dodder, but all these techniques have potential limitations as they may not separate viruses in case of a mixture of viruses. A very interesting and universal technique for an in-depth characterization of a virus is the preparation of infectious clones. The infectious clones, requiring the complete viral genome sequence, offer valuable information about (i) symptomatology for individual viruses and for mixture of viruses, (ii) host range studies and transmissibility, (iii) the assessment of natural or induced mutation/recombination rates in host plants, and (iv) the effects of targeted modifications on virus-host interactions and symptomatology. Mastering virus transmission will also be key to the development of research efforts to understand the potentially positive effects of viruses on plants. Inoculated plants from various species or cultivars can be submitted to biotic or abiotic stresses, and their reaction can be studied in laboratory and/or in greenhouse experiments to provide evidence of beneficial interactions.

### Field surveys

Large-scale surveys, organized on a national or international scale and based on the diagnostic technique(s) developed for the new agent, should be undertaken to evaluate its prevalence and distribution within a given ecosystem (Krenz et al., 2014). These studies will aim to monitor the candidate/demonstrated host plants/vectors. They should be carried out in agricultural ecosystems (fields, commercial orchards or vineyards, nurseries, and germplasm collections), and in natural ecosystems, to enable a holistic characterization of the pathosystems with particular attention to symptomatology. They will complement the preliminary information gained at a smaller scale (See Section Field/Batch Observation) or in the laboratories and greenhouses (See Section Transmission Experiments). The interplay between field surveys and laboratory experiments is particularly important at this stage because it will allow targeting the most appropriate host plant(s) and vector(s), and thus increases knowledge on virus biology.

Large-scale surveys will also provide insights into the genetic variability of a new virus within the surveyed territories. This knowledge will be key for efforts to optimize diagnostic techniques by selecting primers covering the genetic diversity of the species (Massart et al., 2014). In addition, the identification of new variants may bring new fundamental hypotheses on the pathogenicity of the viruses, and these new variants could be characterized under laboratory and greenhouse conditions (See Section Transmission Experiments).

### The specific challenge of virus complexes

The biological characterization of new viruses may be particularly challenging with complex of different viruses, where the combination of two or more viruses can significantly modify their pathogenic potential through synergistic or antagonistic interactions (Martin and Elena, 2009; Syller and Grupa, 2016). Mixture of virus species is indeed very frequent, especially in woody plants like grapevine (Jooste et al., 2015), often leading to unpredictable variations in symptoms, infectivity, accumulation, and/or vector transmissibility. Biological and technological solutions for isolating and determining the role of individual viruses are sometimes possible using selective experimental hosts followed by back-inoculation of “purified” isolates to the original host (Ali and Roossinck, 2008), partial sanitation (Maliogka et al., 2015), infectious clones (Nagyová and Šubr, 2007), or the use of ion-exchange chromatography on monolithic supports to separate viral particles (Ruščić et al., 2015). The biological properties of viruses alone or in mixture can therefore be compared in depth (see Section Field Surveys). The complexity of disease etiology in field conditions might pose additional challenges, as exemplified in the Case Study N°4 on carrot (See Supplementary Material).

## Conclusions

NGS technologies pose new challenges to scientists and to plant health authorities when it comes to analyzing the risks associated with a new virus or pathogen, its potential to spread, or its economic impact and when trying to take a timely decision on whether to let go or destroy contaminated plant materials. As sequencing throughput and bioinformatics analyses are less and less limiting, downstream epidemiology and disease etiology studies will be the obvious bottlenecks in ability to conclude on the biological significance and impact of novel viruses or of complex of different viruses. While exhaustive knowledge on the etiology of a disease or the epidemiology of a virus is very difficult if not impossible to achieve, we propose a framework of experiments and investigations to characterize these newly discovered viruses and to undertake relevant actions in a timely fashion in relation to the context of the finding. In the frame of the risks posed by growing trade and climate change, the aim is to progressively reduce the uncertainties linked with risk management and to help plant health protection authorities decide on the importance of a new virus in a quarantined material or a certified seed stock. However, this decision will also always be influenced by local legislation and the socio-economic and political interpretation of the progressively generated information. Once adopted by authorities and trade, this framework should help to take rationalized decisions on the most relevant actions to take (i.e., confirmation using specific methods, ways to assess impact of a virus in its environment) and potentially prevent “conflicts” between import/export partners.

## Author contributions

All the authors significantly contributed to the writing and editing of the manuscript. SM created the figures. SM and TW coordinated the writing and editing of the manuscript.

## Funding

This article is based upon work from COST Action FA1407 (DIVAS), supported by COST (European Cooperation in Science and Technology).

### Conflict of interest statement

The authors declare that the research was conducted in the absence of any commercial or financial relationships that could be construed as a potential conflict of interest.
