# Supplementary material for: A Framework for the Evaluation of Biosecurity, Commercial, Regulatory, and Scientific Impacts of Plant Viruses and Viroids Identified by NGS Technologies
Source: Front Microbiol. 2017 Jan 24;8:45. doi: 10.3389/fmicb.2017.00045 (PMC5258733; doi:10.3389/fmicb.2017.00045)
Supplement: Supplementary file 1 [file DataSheet1.pdf]

SUPPLEMENTARY MATERIAL – STUDY CASES

Massart *et al*. (2017) A Framework for the Evaluation of Biosecurity, Commercial, Regulatory, and Scientific Impacts of Plant Viruses and Viroids Identified by NGS Technologies. Front. Microbiol. 8:45..

Study Case n°1: Birch leaf roll disease (*Cherry leaf roll virus*)

A disease, slowly causing the trees to decline, was first recognized as a phytopathological issue and described in Finland in 2006 [28]. As the leaf symptoms resemble those of *Cherry leaf roll virus* (CLRV) infection in birches (*Betula* sp.), first investigations were aimed at CLRV detection. CLRV was indeed irregularly detected in diseased *Betula* trees of various species in Finland. Mechanical transmission of viral agents from affected Finnish birch to herbaceous biotest plants failed, whereas other CLRV strains detected in *B. pendula* in Berlin, Germany, or in other hosts were successfully propagated in biotest plants. Transmission by grafting from CLRV-positive *B. pubescens* donor trees from Rovaniemi, Finland, to non-symptomatic *B. pubescens* seedlings was however successful and the grafted seedlings developed similar symptoms to those of donor trees [29]. NGS data obtained by RNA-Seq analysis using leaf tissues from the grafted seedlings as well as from the original donor trees showed that *B. pubescens* seedlings were predominantly infected by a novel putative member of DNA reverse transcribing badnavirus (Baltimore class VII). In addition, a novel Capillo-like virus was also discovered. These new virome data offer new interpretations regarding the cause of the `birch leaf-roll disease´, including the possible involvement of a complex of viruses. The new viruses and their role in disease development need to be characterized to achieve scientifically sound decisions on integrated management of the disease, sustainable forest management, and possibly on the need to reach a regulatory decision about one or more of the viral agents involved.

Massart *et al*. (2017) A Framework for the Evaluation of Biosecurity, Commercial, Regulatory, and Scientific Impacts of Plant Viruses and Viroids Identified by NGS Technologies. Front. Microbiol. 8:45..

Study Case n°2: Nectarine stem pitting

In spring 2013, 5-year old nectarine trees were found exhibiting stunting and extensive stem-pitting symptoms in the USA. The trees had been propagated from budwood of three cultivars imported from France and cleared through the US post-entry quarantine procedure. NGS-based dsRNA sequencing of the trees initially revealed the presence of a new luteovirus that was named Nectarine stem pitting-associated virus (NSPaV) [30]. The presence of NSPaV in the trees was confirmed by RT-PCR, and its genome sequence was completed. However, one symptomatic tree did not test positive for NSPaV. This could indicate a low titer, or variable distribution of the virus in that plant, or a loose association with the symptoms, although NSPaV was the only viral or subviral agent detected. These findings immediately raised a range of questions for the nursery that had initially obtained and supplied the varieties and for the phytosanitary authorities in the USA. In Europe, such a new virus would very likely have been considered as a quarantine agent as it is a “non European virus and virus-like organism of Prunus”, as listed in Annex I A I of European Council Directive 2000/29 which forms the framework of the EU quarantine regulation. In New Zealand and a range of other countries, it would similarly have been considered as falling under quarantine regulations. In an independent work published in 2016 [31], NSPaV was identified, together with a novel Marafivirus named Nectarine virus M (NeVM), in nectarine trees from California exhibiting stem pitting symptoms. Again, the correlation between the presence of these viruses and stem pitting symptoms was imperfect. In addition, all 6 symptomatic trees that were tested displayed coinfection by the two new viral agents, precluding any analysis of their possible individual contribution and suggesting that the symptoms could result from co-infection by NSPaV and NeVM in some specific

nectarine cultivars. Taken together, these results clearly illustrate (i) the power of NGS to reveal viral infection missed by state-of-the-art classical post-quarantine testing, (ii) the complexity of trying to sort out the contribution of (a) viral agent(s) in a given syndrome in mixed infecion, and (iii) the rapid and complex consequences in terms of quarantine regulation, destruction of plant materials and legal action that may derive from such findings.

Massart *et al*. (2017) A Framework for the Evaluation of Biosecurity, Commercial, Regulatory, and Scientific Impacts of Plant Viruses and Viroids Identified by NGS Technologies. Front. Microbiol. 8:45..

Study Case n°3: Grapevine Pinot gris

The discovery of the *Grapevine Pinot gris virus* (GPGV) exemplifies the process of identification and description of a new virus by NGS. It also gives a cross-section of the virome of a woody host and illustrates issues likely to arise when a new virus is found associated with a new disease. GPGV was identified by NGS from grapevine plants showing symptoms of chlorotic mottling and leaf deformations [32]. The viral genome sequence, re-assembled from NGS and classical Sanger sequencing, is homologous to that of the Trichovirus *Grapevine berry inner necrosis virus* (GINV). GPGV viral particles were never observed by electron microscopy in grapevine tissues. The symptoms are reproduced on vine indicators by grafting, and GPGV is transmitted to healthy vines by the mite *Colomerus vitis*. Different studies suggest that GPGV genome variants exist, but that only some of them are able to trigger grapevine leaf mottling and deformation (GLMD) on grapevine. Pathogenic variants accumulate with a higher titer in symptomatic vines [33]. Nevertheless, the majority of grapevines analyzed by NGS disclosed complex viromes, most often including several viral and viroid species. The occurrence of these “background” viruses and viroids in GLMD-infected vines poses questions about the involvement of GPGV in the disease, alone or in combination. More broadly, the same questions generally apply when a new virus is discovered in grapevine. This example underlines how the advent of NGS may in some cases challenge the classical “one virus = one disease” paradigm towards a holistic view in which a new virus may sometimes interact with or disturb the virome, ultimately causing a disease.

Massart *et al*. (2017) A Framework for the Evaluation of Biosecurity, Commercial, Regulatory, and Scientific Impacts of Plant Viruses and Viroids Identified by NGS Technologies. Front. Microbiol. 8:45..

Study Case n°4: Carrot viruses

One of the recent examples of a study on the role of a new virus in complex agroecosystems is the finding of a new carrot virus during a survey of carrot crops displaying external and internal necrosis symptoms in the UK [11]. Viruses known to be common in that geographical region and tested during routine diagnostic (*Parsnip yellow fleck virus*, *Carrot red leaf virus*, *Carrot mottle virus*) were not associated with the disease. NGS results indicated that *Carrot yellow leaf virus* (CYLV) was the most prevalent virus in symptomatic samples, while a novel related Closterovirus, *Carrot closterovirus 1* and other viruses including a new species of Torradovirus (*Carrot torradovirus* 1) were also identified in this survey in both symptomatic and asymptomatic material. A strong correlation between CYLV presence and root necrosis was observed: removing CYLV-infected plants from the population resulted in an estimated 96% reduction of necrosis incidence. However, as pointed out by the authors, a statistical relationship does not prove that CYLV was the sole causal agent of root necrosis. Further targeted diagnostic surveys on a larger sample size, conducted on different sites, and involving different varieties, together with studies on the pathogenicity of CYLV in single or mixed infections, would be required to understand the etiology of the disease and the potential impact of these viruses under field conditions.
